# Effects of Chronic Photobiomodulation with Transcranial Near-Infrared Laser on Seizure Frequency and Brain Metabolomics of Rats with Pilocarpine-Induced Seizures

**DOI:** 10.1007/s12035-025-05175-y

**Published:** 2025-07-14

**Authors:** Fabrizio dos Santos Cardoso, Ricardo Mario Arida, Eduardo Alves da Silva, Ana Carolina Ribeiro de Oliveira, Wagner Ferreira dos Santos, Rodrigo Álvaro Brandão Lopes-Martins, Francisco Gonzalez-Lima, Norberto Cysne Coimbra, Sérgio Gomes da Silva

**Affiliations:** 1 Centro Universitário Redentor/Afya (UniREDENTOR), Itaperuna, RJ Brazil; 2 Hospital Do Câncer de Muriaé, Fundação Cristiano Varella (FCV), Av. Cristiano Ferreira Varella, 555 – Campus, Muriaé, MG 36880-000 Brazil; 3 Hospital São Vicente de Paulo (HSVP), Bom Jesus Do Itabapoana, RJ Brazil; 4 Laboratório de Neurofisiologia, Departamento de Fisiologia, Universidade Federal de São Paulo (UNIFESP), São Paulo, SP Brazil; 5 Departamento de Biologia, Faculdade de Filosofia, Ciências E Letras de Ribeirão Preto da Universidade de São Paulo, Ribeirão Preto, SP Brazil; 6 Universidade Brasil, São Paulo, SP Brazil; 7 Department of Psychology and Institute for Neuroscience, University of Texas at Austin, Austin, TX USA; 8 Laboratório de Neuroanatomia & Neuropsicobiologia, Departmento de Farmacologia, Faculdade de Medicina de Ribeirão Preto da Universidade de São Paulo (FMRP-USP), Ribeirão Preto, SP Brazil; 9 Centro Universitário FAMINAS, Muriaé, MG Brazil

**Keywords:** Laser, Photobiomodulation, Cerebral cortex, Hippocampus, Epilepsy, Seizure frequency, Metabolic profile

## Abstract

Epilepsy is a chronic brain disorder characterized by abnormal and highly synchronous activity of neuronal cells. This condition is often associated with neuronal hyperexcitability and metabolic imbalances in regions such as the neocortex and archicortex (hippocampus). Pharmacological treatment with antiepileptic drugs has been essential in most cases. However, some patients continue to experience seizures despite medication. Consequently, the search for alternative treatments and new therapeutic interventions has garnered significant interest within the medical community. Among these, photobiomodulation (PBM) shows great promise, particularly due to its cerebral and neuroprotective effects. We evaluated and compared the seizure frequency and the neocortical and hippocampal metabolomic profiles of pilocarpine-induced epileptic rats exposed to chronic transcranial photobiomodulation treatment (30 days of treatment) with an 810-nm, 100-mW laser. Our data show that PBM treatment significantly reduced the seizure frequency in rats with pilocarpine-induced seizures. Additionally, significant changes were observed in the metabolomic profiles of the neocortex and hippocampus. In the neocortex, the treatment reduced metabolic pathways associated with excitotoxicity and oxidative stress. In the hippocampus, an increase in phenylalanine concentration was detected. In addition, the reduction in the number of seizures in laser-treated rats with pilocarpine-induced seizures was correlated with lower neocortical lysine concentration. Taken together, our findings indicate that transcranial PBM prevents the increase in seizure frequency in rats with epilepsy and modulates the brain metabolic pathways of epileptic rats.

## Introduction

Epilepsies are neurological diseases characterized by synchronous and unprovoked seizure activity [32, 61]. An epileptic seizure is considered to be a clinical or subclinical disorder of cortical function, generated by a sudden, abnormal, excessive, and disordered electrical discharge of neuronal cells (Fischer et al. 2017), and is classified as having a focal, generalized, or unknown onset [59]. Among the various types of pilocarpine-induced seizures, the most common is temporal lobe epilepsy (TLE. It is characterized by lesions in structures of the limbic system, such as hippocampal sclerosis and cell loss [25].

TLE is accompanied by disturbances in neurotransmitters and energy metabolism. Epileptic seizures are associated with an imbalance in neurotransmission systems, both excitatory and inhibitory [5]. In addition, some studies show that impairments in the metabolic pathways of glycolysis and the activity of the electron transport chain contribute to the onset and worsening of epileptic seizures [3]. In fact, some studies in patients with TLE show a decrease in glucose metabolism and the neuronal marker N-acetyl aspartate in the epileptogenic region during the interictal period occurring between seizures [29, 42]. In animal models of pilocarpine-induced seizures, reductions in glucose utilization are also noted in the hippocampal regions of CA1 and CA3 during epileptic seizures [28]. In addition, animals with epilepsy, such as those induced by either peripheral or intrahippocampal administrations of pilocarpine, exhibit reduced encephalic levels of glutamate, glutathione, γ-aminobutyric acid (GABA), and aspartate [62]. Together these data indicate that, at least in part, epilepsies are associated with dysfunctions in mitochondrial metabolism.

Photobiomodulation (PBM), also called low-level laser therapy, is a non-invasive technique that uses red-to-near-infrared light capable of improving neurological and behavioral functions in several conditions [6–8, 27, 33, 49, 54, 66]. For example, Disner et al. [27] reported that transcranial PBM at 1064-nm wavelength leads to a greater reduction of depression symptoms in participants with better response to attention bias modification. Vargas et al. [66] observed that infrared PBM at 1064 nm, 250 mW/cm^2^, was able to improve the cognitive function and EEG rhythms of older adults with memory complaints. Similar effects have been observed in studies performed with laboratory animals [11, 11, 12, 14, 14, 15, 15, 56]. Salehpour et al. [56] reported that PBM ameliorates cognitive impairment induced by sleep deprivation. In addition, PBM enhanced the antioxidant status and increased mitochondrial activity in the hippocampus of sleep-deprived mice. Our research group noted that PBM improves spatial memory and modulates the levels of inflammatory markers in both the neocortex and hippocampus of aged rats [11, 14, 14, 15]. In addition, chronic PBM restores the brain metabolic pathways of aged rats towards the levels of younger rats [12] and modifies neuronal morphology in the neocortex of rats [15].

Based on the well-documented therapeutic effects of PBM in different neurological conditions [11–15, 20, 41, 48, 54, 56] and the possibility of PBM being a non-pharmacological neurotherapeutic strategy for epilepsy (Cardoso et al., 2022 d), we evaluated whether a chronic transcranial treatment with a laser diode of 810-nm wavelength and 100-mW power can reduce seizure frequency and change the neocortical and hippocampal metabolic profiles in rats with pilocarpine-induced seizures.

## Material and Methods

### Animals

Thirty-seven male Wistar rats (60 days old), from the Ribeirão Preto Campus of the University of São Paulo, were used in this study. The rats were housed at a temperature of 21 ± 2 °C with a 12 h light/dark cycle (lights on from 7 a.m. to 7 p.m.), and food and water were provided ad libitum throughout the experimental period. Rats were randomly distributed in two groups: pilocarpine-induced seizures (E; *n* = 8) and laser pilocarpine-induced seizures (LE; *n* = 9). All procedures were approved by the ethics committee of the University of São Paulo (CEUA-FMRP-USP, process 1030/2021) and all effort was made to minimize animal suffering by the proposals of the International Ethical Guidelines for Biomedical Research (CIOMS 1985) (CIOMS, [22].

### Pilocarpine-Induced Epilepsy Model and Behavioral Analysis

Rats from groups E and LE were submitted to the intraperitoneal (i.p.) treatment with a single dose (350 mg/kg) of pilocarpine hydrochloride (Sigma, ST. Louis, MO Sigma), 30 min after a subcutaneous injection of methylescopolamine (Sigma) in a dose of 1 mg/kg. The administration of methylescopolamine is intended to minimize the peripheral effects of pilocarpine. To terminate seizure activity, diazepam was administered intraperitoneally at a dose of 10 mg/kg, 90 min after the onset of status epilepticus, as established in previous studies using the pilocarpine model [4, 16, 17, 24, 65]. After 4 h of SES, surviving animals were continuously monitored for 24 h using a video system (Intelbras Multi Hd 1120b Dvr 4S VT 120) to quantify the number of spontaneous epileptic seizures (SES). At night time, infrared light was used to allow monitoring. After the first SES was detected, the rats were monitored for two 30-day periods. SES were identified based on clear behavioral manifestations consistent with stage 5 on the Racine scale, including rearing, forelimb clonus, and loss of balance. These criteria were chosen to ensure specificity in detecting generalized convulsive seizures, which are reliably observable through video monitoring. In the first period, all animals remained under observation and with no laser treatment, to analyze the frequency of seizures. In the second period, the animals were randomly divided into a group with laser treatment (LE) and no laser treatment (E).

### Laser Therapy Protocol

Rats from the laser group (LE) were manually immobilized and received the treatment with a laser diode of 810-nm wavelength and 100-mW power for 30 s at each point of application (Therapy XT SN 44526; DMC *Importação e Exportação de Equipamentos Ltda*., São Carlos, SP, Brazil). In the current study, we used five irradiation points in the skullcap (point 1 = AP (anteroposterior) + 4.20 mm and ML (mediolateral) 0.00 mm; point 2 = AP − 3.00 mm and ML − 6.60 mm; 3 = AP − 3.00 mm and ML + 6.60 mm; point 4 = AP 0.00 mm and ML 0.00 mm; point 5 = AP − 5.52 mm and ML 0.00 mm), totaling 15 J of energy, 150 s of irradiation, and fluence of 535.7 J/cm^2^. These laser parameters (810 nm, 100 mW, 30 s per point) have been previously validated by our group and others in experimental studies demonstrating beneficial effects on brain metabolism, inflammation, and cognitive performance in rodents [11, 12, 14]. The use of 810-nm wavelength is further supported by its established capacity to penetrate cortical tissue and stimulate cytochrome c oxidase activity [54]. A Monte Carlo simulation and in vivo measurements of the laser penetration profile in the albino rat brain has been provided by Abdo et al. [1]. It illustrates an exponential light decrease until a 3-mm depth, showing that about 1% light intensity reaches the cortex at 1.5 mm, while less than 0.1% light can reach the hippocampus at 2.5 mm.

Rats from group E received the same procedure as the laser group, however, as placebo treatment (laser turned off). The laser or placebo treatment was maintained throughout the second period (30 days) of the experiment until the rats were anesthetized with i.p. administration of ketamine at 92 mg/kg (Ketamine Agener, União Química Farmacêutica Nacional, São Paulo, Brazil) and xylazine at 9.2 mg/kg (Calmium, União Química Farmacêutica Nacional, São Paulo, Brazil).

### Metabolomics Profile

Twenty-four hours after the last laser therapy session, animals were euthanized by decapitation, and the cerebral neocortex and hippocampus of E (*n* = 8) and LE (*n* = 8) groups were removed and maintained at − 80 °C for analysis of the metabolomic profile. Brain tissue was pulverized using ceramic mortar and pistil under liquid nitrogen. For the extraction procedure, approximately 30 mg of the pulverized material was weighed, and 334 µL of methanol and 166 µL of chloroform (2:1, v/v) were added. The solution was mixed in a vortex for 10 s and sonicated for 5 min. Then, a sample was kept for ~ 15 min in an ice bath. Thereafter, 250 µL of chloroform and 250 µL of Milli-Q water (1:1, v/v) were added, followed by mixing in a vortex for 10 s and centrifugation at 14,000 rpm at 4 °C for 20 min, for subsequent supernatant fraction collection. The procedure was repeated with the precipitated fraction of the supernatant. After this procedure, the supernatant fractions were mixed (~ 0.8 mL), dried using a speed vac (MIVAC DUO), and kept at − 80 °C. For NMR acquisitions, a sample was solubilized in 540 µL of deuterium oxide (D2O, 99.9%; Cambridge Isotope Laboratories Inc., Massachusetts, USA), 60 µL phosphate buffer (0.1 M, pH 7.4), and 0.5 mM TMSP-d4 (3-(trimethylsilyl-2,2′, 3,3′-tetradeuteropropionic acid, Sigma-Aldrich) to produce a final 0.6 mL solution, then transferred to a 5 mm NMR tube (Norell Standard Series 5 mm, Sigma-Aldrich) for immediate data acquisition.

#### NMR Acquisition Spectra

The ^1^H NMR spectra of the samples were acquired using an Agilent Inova 600 spectrometer (Agilent Technologies Inc.™, Santa Clara, USA) from the Brazilian Biosciences National Laboratory (Brazilian Center for Research in Energy and Materials-CNPEM) equipped with a triple resonance cryoprobe and operating at a ^1^H resonance frequency of 599.887 MHz and constant temperature of 298 K (25 °C). A total of 256 or 512 free induction decays, depending on the concentration of metabolites, were collected with 32 k data points at a spectral width of 16 ppm and acquisition time of 4 s. A relaxation delay of 1.5 s was incorporated between the scans, during which a continuous pre-saturation radiofrequency field of water was applied.

#### Identification and Quantification of Metabolites

Data preprocessing, spectral phase, and baseline corrections, as well as the identification and quantification of metabolites present in samples, were performed using Chenomx NMR Suite 8.1 software (Chenomx Inc.™, Edmonton, Canada).

### Statistical Analysis

 Data from experimental neurology experiments were submitted to the Shapiro–Wilk test of normality. For analyzing the frequency of seizures, statistical procedures were conducted using repeated measures of two-way ANOVA. For analyzing the correlation between brain metabolite concentration and seizure frequency, data were also submitted to the Shapiro–Wilk test to verify the normality of the variable distributions. Subsequently, we used either Pearson’s or Kendall’s correlation test, as appropriate. For variables that did not show a normal distribution, outlier values were excluded using Grubbs’ test. If there were no outliers, the data was transformed using the Box-Cox technique to bring the distribution closer to normal. Finally, the *z*-test was used to compare the correlations between the groups. All analyses were performed using the R statistical software v. 4.2.2 [63]. All plots were acquired using the GraphPad Prism (8.0). Data regarding neocortical and hippocampal concentrations of metabolites in the studied groups did not show a Gaussian distribution and were submitted to a non-parametric Mann–Whitney *U* test. Data that followed a Gaussian distribution were represented as the mean and standard error of the mean, while data not normally distributed were represented as median and interquartile. When *p* ≤ 0.05, differences between groups were considered statistically significant.

## Results

### Seizure Frequency

**Fig. 1:**
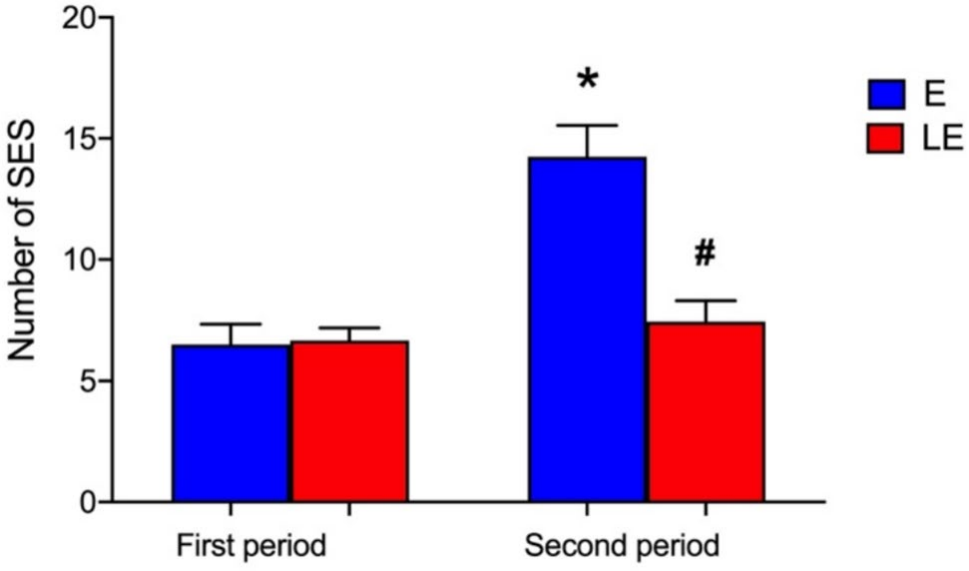
The frequency of SES in rats with pilocarpine-induced seizures was recorded during two different periods of behavioral analysis: 30 days (first period: from the first to the thirtieth day) and 60 days (second period: from the thirty-first to the sixtieth day). The columns represent the mean and the bars are the standard error of the mean of the number of epileptic seizures in groups EL (*n* = 9) and E (*n* = 8). * Significant difference in the number of seizures in the second period compared to the first period observed in rats from group E (*p* < 0.05); ^#^significant difference in the number of seizures in the second period between groups E and EL (*p* < 0.05) (two-way ANOVA with repeated measures, followed by Tukey’s post hoc test)

Seizure frequency was assessed using a video system to quantify the number of SES. Repeated measures ANOVA showed a significant effect of time [*F*_(1,15)_ = 118.3; *p* < 0.001], group [*F*_(1,15)_ = 7.44; *p* = 0.016], and interaction (time × group) (*F*_(1,15)_ = 79.1; *p* < 0.001). When Turkey’s post hoc analysis was performed, no significant difference was detected between the groups studied in the first period (*p* > 0.05). In the second period, one group received PBM treatment (LE group), while the other remained untreated (E group). During this period, we noted that the number of seizures in group E rats increased significantly compared to the first period (*p* < 0.001). However, in the second period, the rats that received laser treatment (LE) had a lower seizure frequency compared to the rats from group E (*p* = 0.002) (Fig. 1). These data show that PBM significantly prevented the increase in seizure frequency in the second period of frequency of SES of the animals submitted to the temporal lobe pilocarpine-induced seizures experimental model.

### Neocortical Metabolomics Profile

**Fig. 2:**
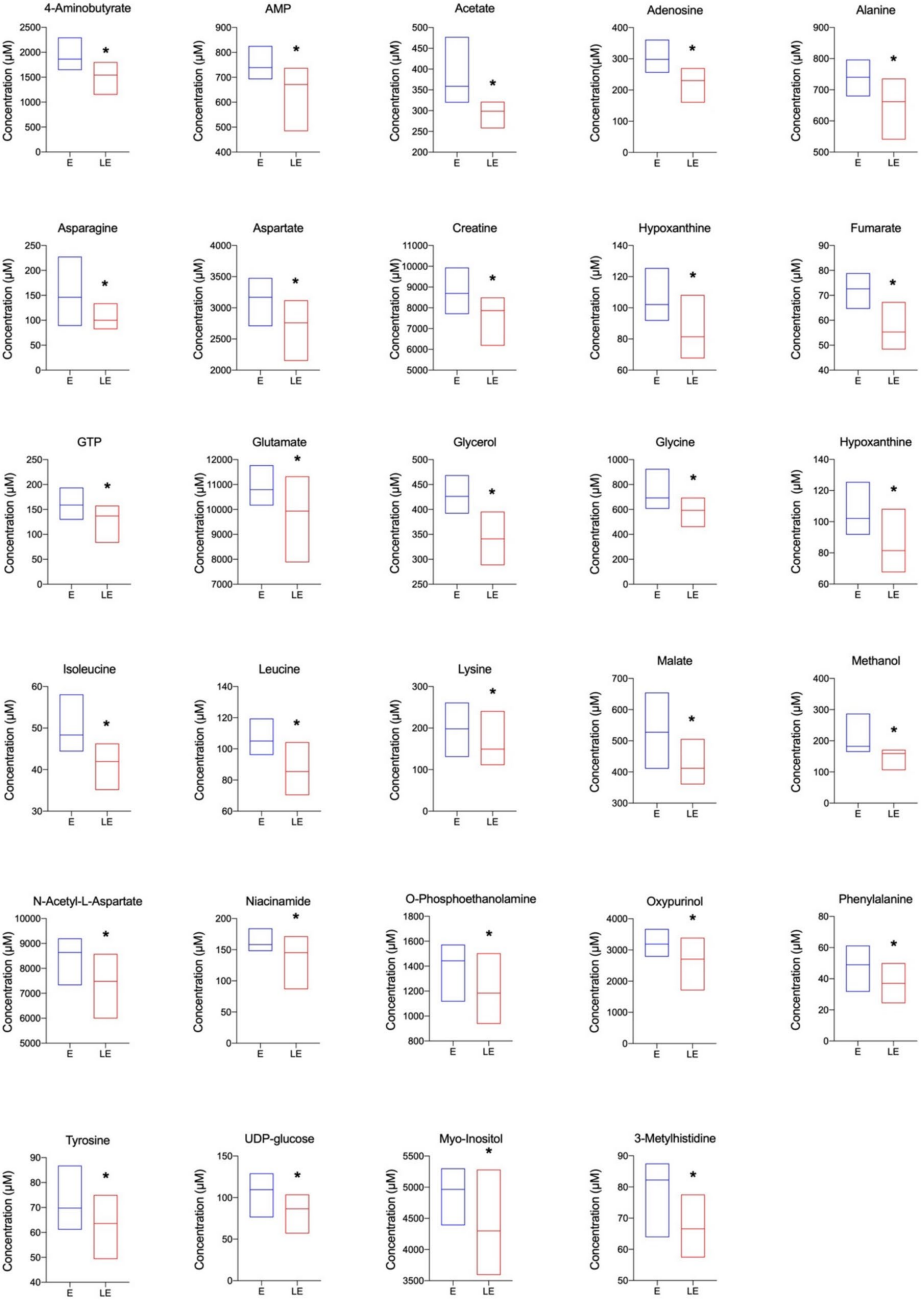
Neocortical concentration of 4-aminobutyrate, 3′,5′-cyclic adenosine monophosphate (AMP), acetate, adenosine, alanine, asparagine, aspartate, creatine, formate, fumarate, guanosine-5′-triphosphate (GTP), glutamate, glycerol, glycine, hypoxanthine, isoleucine, leucine, lysine, malate, methanol, N-acetyl-l-aspartate, niacinamide, O-phosphoethanolamine, oxypurinol, phenylalanine, tyrosine, uridine_diphosphate_(UDP)-glucose, myoinositol, and 3-methylhistidine of the rats in groups E (*n* = 8) and LE (*n* = 8). Data were represented as median 25^th^ and 75^th^ percentiles, minimum and maximum. *Significant (*p* ≤ 0.05) decrease in the concentration of 4-aminobutyrate, AMP, acetate, adenosine, alanine, asparagine, aspartate, creatine, formate, fumarate, GTP, glutamate, glycerol, glycine, hypoxanthine, isoleucine, leucine, lysine, malate, methanol, N-acetyl-l-aspartate, niacinamide, O-phosphoethanolamine, oxypurinol, phenylalanine, tyrosine, UDP-glucose, myoinositol and 3-methylhistidine in the LE group compared to the E group, according to Mann–Whitney test *U* test

In the analysis of the effects of PBM on the neocortical metabolomic profile of rats with pilocarpine-induced seizures, we noted that the rats treated with PBM exhibited a significant reduction in the concentration of 4-aminobutyrate (*p* = 0.007), AMP (*p* = 0.038), acetate (*p* = 0.002), adenosine (*p* = 0.010), alanine (*p* = 0.021), asparagine (*p* = 0.007), aspartate (*p* = 0.038), creatine (*p* = 0.005), formate (*p* = 0.005), fumarate (*p* = 0.001), guanosine-5′-triphosphate (GTP) (*p* = 0.028), glutamate (*p* = 0.028), glycerol (*p* = 0.001), glycine (*p* = 0.007), hipoxanthine (*p* = 0.0466), isoleucine (*p* = 0.003), leucine (*p* = 0.014), lysine (*p* = 0.038), malate (*p* = 0.003), methanol (*p* = 0.001), N-acetyl-l-aspartate (*p* = 0.015), niacinamide (*p* = 0.015), O-phosphoethanolamine (*p* = 0.021), oxypurinol (*p* = 0.028), phenylalanine (*p* = 0.050), tyrosine (*p* = 0.024), uridine diphosphate glucose (UDP) (*p* = 0.015), myo-inositol (*p* = 0.021), and 3-methylhistidine (*p* = 0.016), compared to group E, as shown in Table 1 and Fig. 2.

**Table 1:** Neocortical concentration of metabolites in groups E (n=8) and LE (n=8) (μM). Data are represented as median and interquartile

Metabolites (μM)	E (*n* = 8)	LE (*n* = 8)	*U*	*p*
4-Aminobutyrate	1863.0 (1771.4–2052.8)	1543.0 (1447.7–1757.8)	7.00	0.007*
4-Pyridoxate	46.0 (41.7–50.1)	35.7 (32.7–32.7)	17.00	0.130
ADP	228.0 (208.8–259.7)	194.6 (182.8–207.6)	14.00	0.065
AMP	739.0 (710.9–767.7)	671.2 (604.5–731.1)	12.00	0.038*
Acetate	358.6 (355.6–375.0)	298.6 (273.0–317.9)	2.00	< 0.001*
Adenosine	298.2 (266.9–318.6)	230.1 (219.2–265.8)	8.00	0.010*
Alanine	740.2 (701.8–752.7)	662.0 (599.8–696.7)	10.00	0.021*
Ascorbate	1904.0 (1814.1–1982.2)	1773.0 (1520.5–1938.6)	21.00	0.279
Asparagine	146.1 (137.3–153.4)	100.0 (97.9–126.1)	7.00	0.007*
Aspartate	3170.0 (2947.3–3375.3)	2760.0 (2600.6–2886.2)	12.00	0.038*
Betaine	31.7 (30.5–35.5)	30.2 (26.1–32.0)	20.50	0.244
Choline	222.6 (202.1–242.8)	206.4 (196.3–215.7)	18.50	0.170
Creatine	8693.0 (8462.0–8971.8)	7866.0 (7058.8–8191.6)	6.00	0.005*
Creatine phosphate	40.8 (34.2–53.8)	37.80 (35.2–46.0)	30.00	0.855
Creatinine	32.3 (28.3–37.9)	28.6 (23.8–32.7)	21.00	0.279
Ethanol	131.8 (108.3–150.3)	105.3 (83.3–115.6)	14.00	0.060
Formate	93.4 (87.0–98.0)	65.0 (59.7–74.6)	6.00	0.005*
Fumarate	72.6 (68.0–75.6)	55.3 (54.3–57.4)	2.00	< 0.001*
GTP	158.8 (144.0–178.1)	137.1 (106.7–145.4)	11.00	0.028*
Glutamate	10,794.0 (10,341.2–10,890.5)	9934.0 (9266.7–10,109.5)	10.00	0.021*
Glutamine	4638.0 (4518.2–4826.3)	4467.0 (4039.2–4718.4)	21.00	0.279
Glutathione	748.6 (704.2–783.7)	661.7 (543.8–711.9)	14.00	0.065
Glycerol	426.1 (403.5–447.5)	340.9 (319.2–365.3)	2.00	< 0.001*
Glycina	692.8 (669.3–738.5)	592.9 (555.2–634.2)	7.00	0.007*
Histidine	47.8 (43.3–50.7)	39.6 (34.2–42.2)	17.00	0.130
Hypoxanthine	102.1 (100.0–109.9)	81.4 (77.9–102.9)	13.00	0.0466*
IMP	139.4 (130.4–152.7)	121.4 (101.9–139.2)	16.00	0.105
Inosine	140.5 (135.3–148.8)	133.1 (115.9–145.6)	23.00	0.382
Isoleucine	48.4 (47.2–50.0)	42.0 (38.8–44.3)	3.00	< 0.001*
Lactate	11,094.0 (10,802.9–11,491.2)	9804.0 (8975.0–10,924.1)	15.00	0.083
Leucine	105.0 (100.0–110.2)	85.4 (78.7–97.9)	8.00	0.001*
Lysine	198.1 (185.9–227.7)	149.4 (128.2–167.3)	12.00	0.038*
Malate	527.7 (506.3–564.3)	411.8 (380.0–457.5)	5.00	0.003*
Methanol	182.3 (174.1–200.1)	159.2 (123.8–165.5)	3.00	0.001*
N-Acetyl-l-aspartate	8639.0 (8325.0–8801.6)	7482.0 (7027.5–8239.9)	9.00	0.015*
Niacinamide	158.3 (153.4–158.3)	145.2 (137.9–153.8)	9.00	0.014*
O-Phosphocholine	264.9 (254.9–296.3)	286.4 (214.7–281.5)	18.00	0.161
O-Phosphoethanolamine	1443.0 (1389.6–1542.6)	1184.0 (1119.0–1305.5)	10.00	0.021*
Oxypurinol	2704.0 (2911.3–3372.4)	3189.0 (2553.5–2885.1)	11.00	0.028*
Phenylalanine	49.0 (44.7–54.2)	37.0 (32.9–43.4)	12.50	0.040*
Pyruvate	10.1 (9.2–14.9)	9.0 (5.9–12.8)	22.50	0.342
Succinate	256.7 (247.9–294.4)	314.3 (306.7–333.5)	16.00	0.105
Taurine	5635.0 (5924.1–6472.4)	6358.0 (4906.1–6391.3)	22.00	0.328
Threonine	352.1 (321.1–409.3)	358.0 (293.6–388.9)	28.00	0.721
Tyrosine	69.8 (68.4–74.8)	63.6 (57.6–66.3)	10.00	0.020*
UDP-glucose	109.6 (103.1–119.1)	86.55 (83.8–95.5)	9.00	0.015*
Uridine	89.0 (82.2–97.7)	80.7 (73.5–96.2)	23.00	0.382
Valine	107.8 (104.1–114.5)	97.1 (86.7–111.8)	19.00	0.195
Myo-Inositol	4965.5 (4780.1—5095.1)	4299.0 3793.2–4472.1)	10.00	0.021*
sn-Glycero-3-phosphocholine	624.5 (601.0–685.3)	563.0 (518.0–734.1)	26.00	0.574
3-Metylhistidine	82.3 (74.8–84.2)	66.6 (63.6–71.4)	8.50	0.011*

* Significant difference (*p* 0.05) when compared to the control, according to the Mann-Whitney U-test

#### Neocortical Metabolic Pathways Altered by PBM in Rats with Pilocarpine-Induced Seizures

After observing the differences between the studied groups, it was possible to analyze the impacted metabolic pathways through the metabolite set enrichment analysis (MSEA). This analysis was performed with metabolites that exhibited significant changes among the groups.

**Fig. 3:**
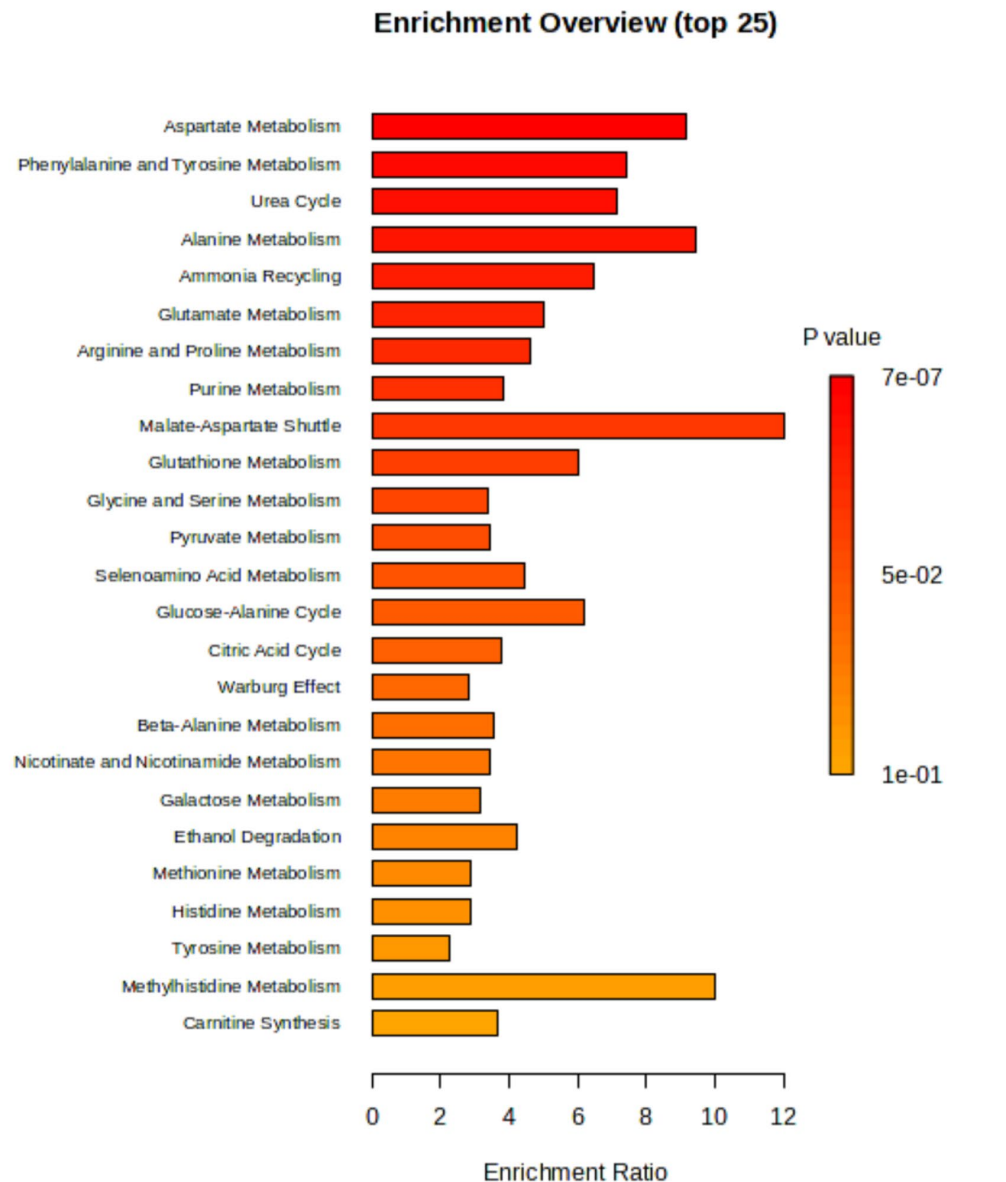
Neocortical metabolic pathways altered by PBM performed in rats with pilocarpine-induced seizures, generated by the online metabolic platform MetaboAnalyst

When we analyzed the neocortical metabolic pathways altered by PBM in rats with pilocarpine-induced seizures, we observed a significant decrease in the pathways of aspartate metabolism (*p* < 0.0001), phenylalanine and tyrosine metabolism (*p* = 0.0127), urea cycle (*p* = 0.0127), alanine metabolism (*p* = 0.0127), ammonia recycling (*p* = 0.0127), and glutamate metabolism (*p* = 0.004) (Fig. 3).

### Hippocampal Metabolomics Profile

**Fig. 4:**
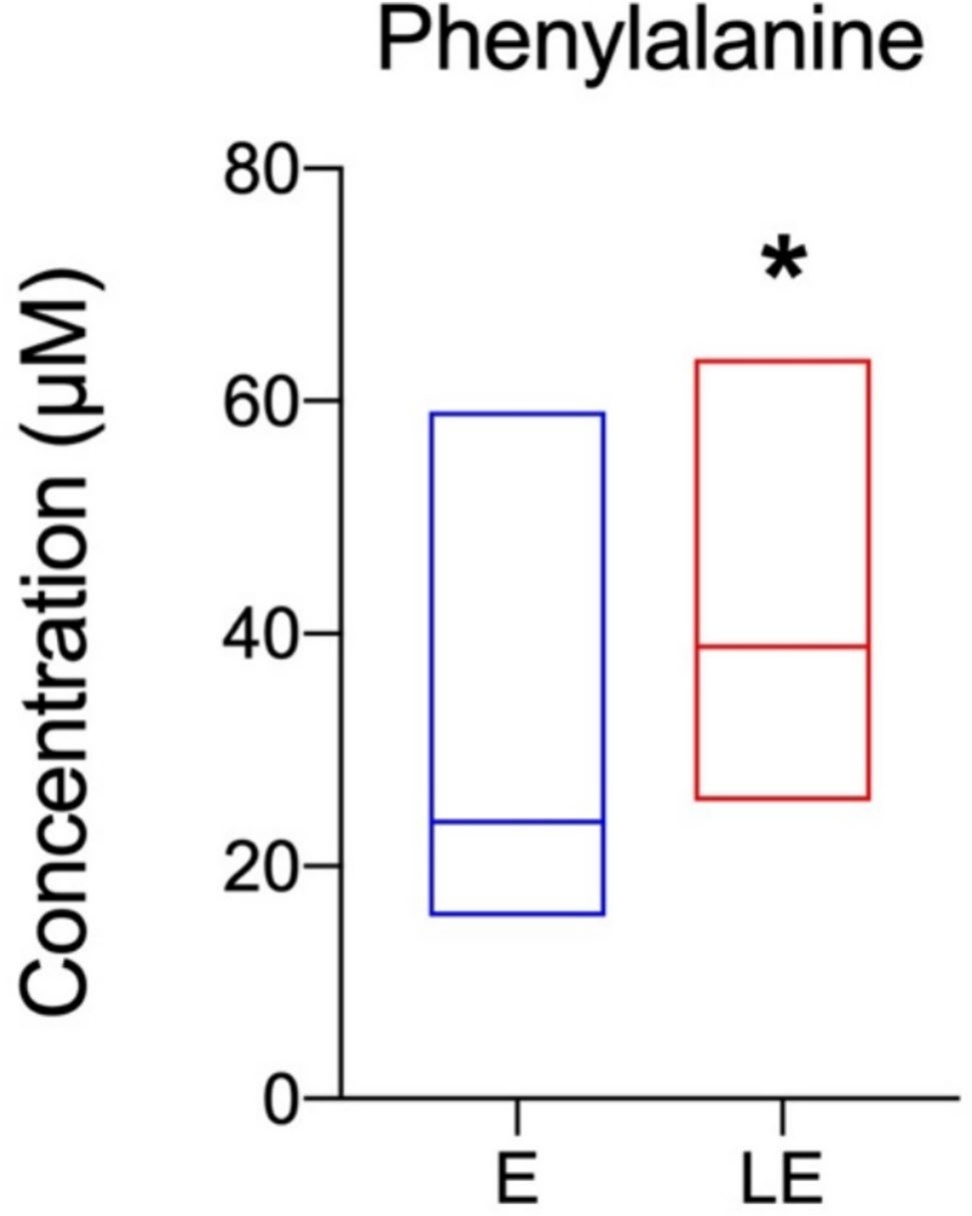
Concentration in µM of phenylalanine in the hippocampus of rats from E (*n* = 8) and LE (*n* = 7) groups; data were represented as median 25^th^ and 75^th^ percentiles, minimum and maximum. *Significant difference (*p* < 0.05) in comparison to the control group, according to the Mann–Whitney *U* test

In the analysis of the effects of PBM on the hippocampal metabolomic profile of rats with pilocarpine-induced seizures, the rats treated with PBM exhibited an increase in the concentration of phenylalanine (*p* = 0.040) compared to group E (Table 2 and Fig. 4). We could not perform analyses of the hippocampal metabolic pathways because we observed a difference only in phenylalanine concentration.

**Table 2:** Hippocampal concentration of metabolites in groups E (n=8) and LE (n=7) (μM). Median and interquartiles

Metabolite (μM)	E (*n* = 8)	LE (*n* = 7)	*U*	*p*
4-Aminobutyrate	1639.3 (1217.3–1855.2)	1824.4 (1579.7–1875.0)	22.00	0.536
4-Pyridoxate	38.9 (27.0–51.3)	40.0 (26.0–61.9)	27.00	0.955
ADP	156.8 (137.5–166.5)	162.1 (131.9–204.2)	21.00	0.463
AMP	405.7 (372.1–467.7)	444.8 (408.5–530.9)	21.00	0.463
Acetate	395.0 (341.6–473.3)	482.6 (381.9–512.2)	16.00	0.189
Adenosine	231.4 (164.0–275.0)	260.6 (239.5–368.5)	17.00	0.232
Alanine	711.4 (603.5–878.0)	862.2 (784.8–920.9)	16.00	0.189
Ascorbate	1403.0 (1097.4–1865.5)	1844.8 (1635.1–1924.3)	18.00	0.281
Asparagine	104.9 (74.3–144.3)	150.0 (142.3–201.6)	12.00	0.072
Aspartate	1564.3 (1261.3–2166.6)	1865.2 (1817.5–1950.0)	22.00	0.536
Betaine	30.8 (23.7–37.3)	35.6 (32.2–37.6)	20.00	0.397
Choline	146.5 (113.5–235.1)	202.2 (181.5–241.6)	18.00	0.281
Creatine	6867.1 (5567.1–8307.8)	7751.1 (7607.9–8120.7)	19.00	0.336
Creatine phosphate	34.3 (24.2–41.0)	39.1 (38.3–41.0)	17.00	0.232
Creatinine	28,6 (15.3–37.6)	34.9 (34.1–36.4)	20.00	0.395
Ethanol	188.9 (146.9–262.8)	179.3 (121.1–264.0)	26.00	0.867
Formate	111.8 (105.4–124.4)	131.0 (88.7–171.5)	23.00	0.613
Fumarate	45.7 (36.3–63.6)	62.1 (54.4–70.9)	18.00	0.281
GTP	101.4 (89.7–129.0)	126.7 (117.6–129.5)	16.00	0.189
Glutamate	7302.9 (6263.7–8923.5)	9437.2 (8990.9–9674.0)	13.00	0.094
Glutamine	3382.4 (2407.7–4300.5)	4133.3 (3913.2–4187.5)	22.00	0.536
Glutathione	519.9 (375.9–618.4)	644.4 (524.4–659.1)	18.00	0.281
Glycerol	369.8 (281.5–449.1)	496.6 (372.2–564.6)	14.00	0.121
Glycine	591.0 (406.5–787.3)	706.7 (658.1–772.3)	21.00	0.463
Histidine	25.5 (14.2–40.7)	35.6 (23.2–39.9)	21.00	0.463
Hypoxanthine	81.3 (61.7–132.2)	118.2 (97.5–139.0)	16.00	0.189
IMP	109.6 (74.9—153.6)	117.4 (103.6–129.0)	26.00	0.867
Inosine	170.0 (133.3–193.6)	227.8 (152.0–247.7)	22.00	0.536
Isoleucine	31.8 (30.9–40.2)	43.5 (41.6–47.8)	14.50	0.128
Lactate	10,731.4 (7997.8–13,151.0)	11,797.8 (10,861.7–12,123.2)	23.00	0.613
Leucine	74.2 (68.2–87.5)	100.0 (83.2–109.9)	15.50	0.159
Lysine	130.6 (102.9–171.3)	178.3 (152.6–214.3)	15.00	0.152
Malate	324.3 (253.4–507.5)	482.8 (400.3–515.0)	16.00	0.189
Methanol	266.7 (250.5–287.9)	258.1 (239.7–452.2)	25.00	0.779
N-Acetyl-l-aspartate	5832.6 (4699.0–7055.6)	6927.9 (6765.1–7227.5)	17.00	0.232
Niacinamide	117.3 (99.7–144.6)	139.5 (126.3–164.8)	22.00	0.536
O-Phosphocholine	282.8 (194.9–334.4)	310.3 (276.0–328.9)	22.00	0.516
O-Phosphoethanolamine	1077.0 (851.7–1370.1)	1241.4 (1212.2–1267.9)	21.00	0.463
Oxypurinol	2134.3 (1899.0–2716.3)	2520.7 (2304.5–2671.6)	21.00	0.463
Phenylalanine	23.8 (19.3–30.0)	38.9 (31.1–42.0)	10.00	0.040*
Pyruvate	13.2 (9.6–17.5)	14.0 (12.7–17.7)	23.00	0.592
Succinate	177.5 (169.7–214.7)	211.6 (177.3–226.0)	23.00	0.613
Taurine	5010.0 (4018.9–6121.7)	5946.7 (5619.2–6418.4)	17.00	0.232
Threonine	273.5 (193.8–337.2)	336.4 (290.0–357.2)	19.00	0.336
Tyrosine	53.1 (39.7–80.5)	66.7 (54.0–73.0)	21.00	0.463
UDP-glucose	73.2 (60.6–85.0)	81.8 (76.3–87.3)	17.00	0.232
Uridine	64.3 (51.2–94.6)	83.3 (73.0–110.3)	14.50	0.128
Valine	91.1 (76.9–99.6)	103.4 (97.7–108.3)	16.00	0.189
Myo-Inositol	4860.0 (4033.9–5946.7)	5588.9 (5383.8–5683.7)	21.00	0.463
sn-Glycero-3 phosphocholine	481.8 (442.3–655.4)	660.5 (629.8–690.2)	14.00	0.121
3-Metylhistidine	42.0 (38.2–50.2)	51.5 (41.5–69.2)	20.00	0.397

* Significant difference (*p* 0.05) when compared to the control, according to the Mann-Whitney U-test

### Correlation Between the Frequency of SES and Metabolic Alterations

**Fig. 5:**
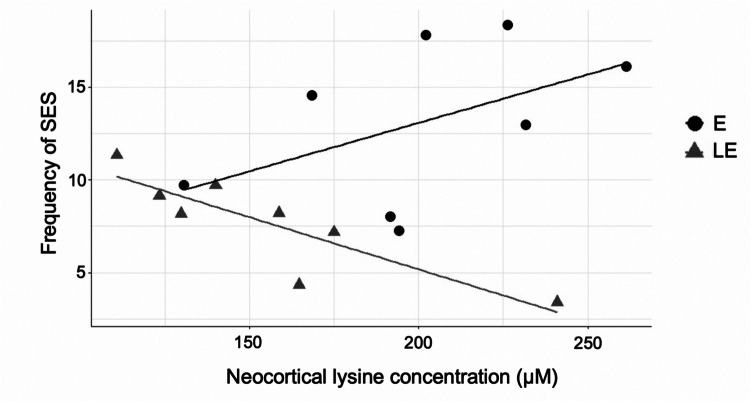
Correlation between neocortical lysine concentration and frequency of SES in groups EL (*n* = 8) and E (*n* = 8)

We used the Pearson’s correlation test to identify if brain metabolites are correlated with the frequency of SES. Our data showed a significant relation between neocortical lysine and SES. In rats with pilocarpine-induced seizures treated with PBM, a reduction in number of SES was significantly related to a lower concentration of neocortical lysine (*r* = − 0.86; *p* = 0.0059), as shown in Fig. 5.

## Discussion

Our findings indicate that transcranial PBM significantly prevented the increase of SES frequency and broadly modulated the neocortical metabolomics profile of rats with pilocarpine-induced seizures. In the hippocampus, only a significant increase in phenylalanine concentration was detected, suggesting a very limited PBM effect likely due to the ten times lower dose of light intensity reaching this deeper region as compared to the neocortex [1].

The PBM treatment had beneficial effects on seizure occurrence in rats with pilocarpine-induced seizures. Rats submitted to PBM treatment with a laser diode of 810-nm wavelength and 100-mW power during 30 days were able to significantly decrease the number of seizures compared to non-treated rats. Our results are in accordance with studies that showed that PBM treatment reduces the number of seizures in epileptic rats [37, 64, 68]. For example, PBM treatment with 780-nm wavelength for 24 sessions reduced seizure duration in rats with stroke-induced epilepsy [68]. Tsai et al. [64] noted that PBM at 808-nm wavelength attenuates pentylenetetrazole-induced acute seizure and convulsive status epilepticus in peripubertal rats. In the study by Hong et al. [37], the PBM using an 825-nm wavelength laser attenuated pilocarpine‐induced acute seizure. Despite this previous work, our study is the first to investigate the effects of PBM on the frequency of recurrent seizures in rats with pilocarpine-induced seizures.

In our study, the attenuation of the increase in the frequency of SES in rats treated with PBM was accompanied by a significant decrease in the neocortical metabolic pathways of aspartate metabolism, phenylalanine, and tyrosine metabolism, urea cycle, alanine metabolism, ammonia recycling, and glutamate metabolism. Interestingly, the urea cycle is initiated by N-acetylglutamate synthase (NAGS), which catalyzes the conversion of glutamate into N-acetyl-l-Glutamate [9]. Urea cycle disorders are characterized by altered levels of citrulline, ornithine, and arginine, depending on the site of the metabolic blockade. This impaired urea cycle leads to hyperammonemia, which can be detoxified by the formation of glutamine and alanine (non-toxic ammonia transporters) from glutamate and pyruvate, respectively. Toxic hyperammonemia results in the typical clinical phenotype characterized by headaches, lethargy, and seizures [2]. NAGS deficiency impairs the urea cycle, resulting in hyperammonemia, increased concentrations of glutamine, and hypocitrullinemia [58]. NAGS deficiency also induces excess glutamate [10, 55].

Supporting the potential therapeutic effects of PBM, the decrease in aspartate and glutamate, two excitatory neurotransmitters, may be directly related to the reduction of neuronal hyperexcitability, a characteristic of epilepsy. Experimental studies using animal models pilocarpine-induced seizures show that excess glutamate and aspartate in the brain are associated with uncontrolled neuronal excitation, mainly through the activation of N-methyl-d-aspartate (NMDA) and α-amino-3-hydroxy-5-methyl-4-isoxazolepropionic acid (AMPA)/kainate glutamatergic receptors, which are fundamental for rapid synaptic transmission and neuroplasticity [35, 43, 47]. NMDA receptor hyperactivity, particularly in conditions of excess glutamate, can lead to excitotoxicity and facilitate the propagation of epileptic seizures, as observed in studies with increased expression of these proteins in the hippocampus during seizures [19].

Reducing glutamate levels can therefore decrease synaptic excitability, limiting NMDA receptor activation and, consequently, reducing seizure susceptibility [43]. Experimental studies have shown that interventions that modulate glutamate availability, such as the use of glutamatergic receptor antagonists, can inhibit epileptic activity in animal models of pilocarpine-induced seizures, reinforcing the importance of maintaining balanced levels of this neurotransmitter [31, 39, 47]. Similarly, aspartate, which can also act as an agonist of NMDA receptors, plays a role in maintaining synaptic excitability. The decrease in aspartate levels observed in experimental models after seizures may contribute to a neuronal stabilization effect, creating an environment less susceptible to excessive neuronal firing, characteristic of epileptic seizures [39]. Thus, modulating these neurotransmitters is essential for reducing hyperexcitability in epileptic contexts.

In the hippocampus, our data show that laser treatment significantly increased the concentration of phenylalanine. This finding is particularly interesting because phenylalanine is an essential amino acid that serves as a precursor for the synthesis of neurotransmitters such as dopamine, norepinephrine, and epinephrine. Reduced levels of phenylalanine are associated with impairments in the production of these neurotransmitters, affecting neurotransmission and neuronal homeostasis [21, 67]. Disruptions in phenylalanine metabolism and biosynthesis pathways cause depressive symptoms and alterations in functional brain networks [45, 71]. The reduction in phenylalanine levels may be related to the randomization trend of β−1 brain waves (12–15 Hz frequency) in the functional brain network of depressive patients [71]. Furthermore, these metabolic alterations may be associated with the cognitive and behavioral deficits observed in mice [67].

Our findings showed an opposite general pattern of changes in metabolite concentrations in the neocortical and hippocampal regions of rats with pilocarpine-induced seizures in response to PBM. While we observed a decrease in metabolite concentrations in the neocortex, there was an increase in metabolite concentrations in the hippocampus of laser-treated rats. These divergent effects between the two brain regions are possibly related to differences in regional physiology, the function of the structures involved, and cellular responses to laser stimulation. For example, in the neocortex, the reduction in the concentration of several metabolites, including glutamate, aspartate, and other metabolites associated with energy metabolism, may be involved in the ability of the PBM procedure to regulate neuronal excitability and modulate excitatory neurotransmitter levels in the brain. Additionally, the neocortex is a key region for the generation and propagation of epileptic activities, often affected by excessive glutamatergic activity, one of the main mechanisms triggering epileptic seizures [5]. The reduction in glutamate and aspartate levels suggested by our data may indicate that PBM promotes a local inhibitory effect, reducing excessive excitation and, consequently, the vulnerability of the neocortex to epileptic seizures.

Additionally, PBM has been associated with enhanced mitochondrial function, neocortical oxygenation, and improved cellular metabolic capacity [12, 36, 56]. In the neocortex, this improvement in mitochondrial efficiency may lead to increased utilization of metabolites as energy substrates, contributing to their reduced concentration in the encephalic tissue. The heightened energy demand during neuronal stabilization after PBM treatment may result in the rapid metabolism of molecules such as glutamate and aspartate, directing them into pathways that promote the restoration of excitatory/inhibitory balance in cerebral cortexes.

On the other hand, the increase in phenylalanine concentration observed in the hippocampus suggests a different dose–response to PBM in this region. The very low dose of light penetrating the hippocampus may contribute to a PBM biphasic (hormetic) dose–response, by which low light doses in the hippocampus have different metabolic effects than higher light doses in the cortex [69]. The hippocampus plays a crucial role in regulating inhibitory control of epileptic seizures [18, 50]. For instance, the elevation in phenylalanine concentration contributes to mitochondrial stability and the activation of the AMP-activated protein kinase AMPK signaling pathway, increasing energy reserves [70]. This phenomenon possibly occurs as a compensatory mechanism to sustain the metabolic activity required for neuroprotection and recovery following episodes of hyper-excitability [34, 46]. These data are relevant because the hippocampus is a region frequently damaged during epileptic seizures in temporal lobe epilepsy [44, 60].

Our data suggest that higher-dose PBM promotes increased efficiency in the utilization of metabolites in the neocortex. Meanwhile, in the hippocampus, lower-dose PBM facilitates the synthesis of molecules that promote an adaptive response to metabolic stress caused by seizures. The difference in PBM effects between the neocortex and hippocampus may also be influenced by mitochondrial distribution and the cellular heterogeneity of these regions [52]. The neocortex, being a region with a high density of synapses and a greater diversity of cell types, may respond to PBM with a greater mobilization of its energy resources to maintain neuronal homeostasis. In contrast, the hippocampus, which is a region more vulnerable to neurodegeneration and energy dysfunction in epilepsy cases [40], may direct the PBM response toward the preservation and replenishment of substrates necessary to sustain its long-term function. Additionally, the amount of laser light reaching the neocortex is larger than the amount reaching the deeper hippocampus in rats [69], suggesting that a different PBM dose could contribute to the different regional responses. These findings highlight the complexity of the mechanisms of action of PBM in the brain and suggest that this non-pharmacological therapy can be used in a targeted manner, depending on the metabolic and functional needs of each encephalic region in specific neurological conditions, such as epilepsy.

Additionally, neuronal populations and mitochondrial profiles differ between the neocortex and hippocampus, which could lead to region-specific metabolic responses to PBM. For instance, hippocampal neurons display distinct mitochondrial morphology and distribution compared to cortical neurons, reflecting region-dependent bioenergetic demands and plasticity [30]. Moreover, differences in neurovascular coupling and oxygenation between these regions may influence their responsiveness to mitochondrial stimulation by PBM [57]. The neocortex is also critically involved in seizure propagation and maintenance. Intracranial recordings in both animal models and human patients with drug-resistant epilepsy have shown that ictal discharges often originate in deep cortical layers and propagate through supragranular circuits [53], suggesting that modulating neocortical activity could yield therapeutic benefits even when the primary epileptogenic focus lies elsewhere. Finally, while only one metabolite (phenylalanine) was significantly altered in the hippocampus, this change may represent an early or subtle neurochemical response. Elevated phenylalanine levels have been shown to impact dendritic branching and synaptic density in cortical neurons [38], supporting the hypothesis that its modulation may precede broader metabolic remodeling in the hippocampal region.

Another finding of our study was the negative correlation between cortical lysine concentration and the frequency of epileptic seizures in PBM-treated animals. We observed that the reduction in seizure frequency in treated rats was associated with lower cortical lysine levels. This finding suggests that specific metabolic pathways involving lysine may contribute to neuroprotection and the reduction of neuronal excitability.

Lysine is an essential amino acid with important functions in brain metabolism, such as participation in protein synthesis and regulation of metabolic pathways related to energy production and neurotransmitters [23, 26, 51]. The neocortical lysine levels in PBM-treated animals may be related to metabolic efficiency in the telencephalon, promoting a state of reduced vulnerability to seizures. Therefore, PBM may act not only by reducing seizure frequency but also by reestablishing metabolic and functional neocortical homeostasis.

One limitation of our study is the exclusive use of the pilocarpine-induced model of temporal lobe epilepsy. While this model replicates key clinical and neuropathological features of epilepsy, it does not represent the full spectrum of seizure etiologies. Additionally, seizure frequency was assessed exclusively through video monitoring without concurrent EEG recordings. Although video analysis allows for the reliable detection of convulsive seizures, it cannot capture subclinical or electrographic seizures, nor provide spectral data such as frequency, amplitude, and rhythmicity. Furthermore, the absence of electrophysiological and histological analyses limits our ability to draw mechanistic conclusions about the effects of PBM. While our findings provide important preliminary insights, we acknowledge the exploratory nature of this analysis. The absence of EEG recordings, functional validation, and the exclusive use of the pilocarpine model limits mechanistic interpretations. As such, the proposed links between metabolite shifts and seizure modulation should be interpreted with caution. Future studies incorporating electrophysiology, immunohistochemistry, molecular biology, and transcriptomics will be essential to experimentally validate the hypotheses raised and deepen our understanding of the therapeutic mechanisms of PBM in epilepsy.

Taken together, our data indicate that chronic transcranial PBM prevents the increase in seizure frequency in rats with pilocarpine-induced seizures and modulates the brain metabolic pathways of rats with pilocarpine-induced seizures. In addition, seizure frequency was modulated by the neocortical concentration of lysine.

## Data Availability

No datasets were generated or analysed during the current study.

## References

[1] Abdo A, Ersen A, Sahin M (2013) Near-infrared light penetration profile in the rodent brain. J Biomed Opt 18(7):075001–075001 23831713 10.1117/1.JBO.18.7.075001 PMC3701316

[2] Ah Mew N, Simpson KL, Gropman AL, Lanpher BC, Chapman KA, Summar ML (1993) Urea cycle disorders overview. Gene Reviews

[3] Alvestad S, Hammer J, Qu H, Håberg A, Ottersen OP, Sonnewald U (2011) Reduced astrocytic contribution to the turnover of glutamate, glutamine, and GABA characterizes the latent phase in the kainate model of temporal lobe epilepsy. J Cereb Blood Flow Metab 31(8):1675–1686 21522161 10.1038/jcbfm.2011.36 PMC3170943

[4] Arida RM, Scorza FA, dos Santos NF, Peres CA, Cavalheiro EA (1999) Effect of physical exercise on seizure occurrence in a model of temporal lobe epilepsy in rats. Epilepsy Res 37(1):45–52 10515174 10.1016/s0920-1211(99)00032-7

[5] Avoli M, De Curtis M, Gnatkovsky V, Gotman J, Köhling R, Lévesque M, Williams S (2016) Specific imbalance of excitatory/inhibitory signaling establishes seizure onset pattern in temporal lobe epilepsy. J Neurophysiol 115(6):3229–3237 27075542 10.1152/jn.01128.2015 PMC4946603

[6] Barrett DW, Gonzalez-Lima F (2013) Transcranial infrared laser stimulation produces beneficial cognitive and emotional effects in humans. Neuroscience 230:13–23 23200785 10.1016/j.neuroscience.2012.11.016

[7] Barrett DW, Davis RE, Siegel-Ramsay JE, Bichlmeier A, Almeida JR, Gonzalez-Lima F (2025) Cognitive improvement and prefrontal network interactions in individuals with remitted bipolar disorder after transcranial infrared laser stimulation. Front Psych 16:1547230 10.3389/fpsyt.2025.1547230 PMC11822565 39950176

[8] Blanco NJ, Maddox WT, Gonzalez-Lima F (2017) Improving executive function using transcranial infrared laser stimulation. J Neuropsychol 11(1):14–25 26017772 10.1111/jnp.12074 PMC4662930

[9] Caldovic L, Morizono H, Panglao MG, Gallegos R, Yu X, Shi D, Tuchman M (2002) Cloning and expression of the human N-acetylglutamate synthase gene. Biochem Biophys Res Commun 299(4):581–586 12459178 10.1016/s0006-291x(02)02696-7

[10] Caldovic L, Morizono H, Panglao MG, Cheng SF, Packman S, Tuchman M (2003) Null mutations in the N-acetylglutamate synthase gene associated with acute neonatal disease and hyperammonemia. Hum Genet 112:364–368 12594532 10.1007/s00439-003-0909-5

[11] Cardoso FDS, Mansur FCB, Araújo BHS, Gonzalez-Lima F, Gomes da Silva S (2022) Photobiomodulation improves the inflammatory response and intracellular signaling proteins linked to vascular function and cell survival in the brain of aged rats. Mol Neurobiol 59(1):420–428 34708330 10.1007/s12035-021-02606-4

[12] Cardoso FDS, Dos Santos JCC, Gonzalez-Lima F, Araújo BHS, Lopes-Martins RÁB, Gomes da Silva S (2021) Effects of chronic photobiomodulation with transcranial near-infrared laser on brain metabolomics of young and aged rats. Mol Neurobiol 58(5):2256–2268 33417219 10.1007/s12035-020-02247-z

[13] Cardoso FD, de Souza Oliveira Tavares C, Araujo BH, Mansur F, Lopes-Martins RA, Gomes da Silva S (2022) Improved spatial memory and neuroinflammatory profile changes in aged rats submitted to photobiomodulation therapy. Cell Mol Neurobiol 42(6):1875–1886 10.1007/s10571-021-01069-4 PMC11421705 33704604

[14] Cardoso FDS, Serra FT, Coimbra NC, Gonzalez-Lima F, Gomes da Silva SG (2022) Transcranial photobiomodulation changes neuronal morphology in the cerebral cortex of rats. Neurosci Lett 781:136681 35569700 10.1016/j.neulet.2022.136681

[15] Cardoso FDS, Gonzalez-Lima F, Coimbra NC (2022) Mitochondrial photobiomodulation as a neurotherapeutic strategy for epilepsy. Front Neurol 13:873496 35785362 10.3389/fneur.2022.873496 PMC9243228

[16] Cavalheiro EA, Leite JP, Bortolotto ZA, Turski WA, Ikonomidou C, Turski L (1991) Long-term effects of pilocarpine in rats: structural damage of the brain triggers kindling and spontaneous I recurrent seizures. Epilepsia 32(6):778–782 1743148 10.1111/j.1528-1157.1991.tb05533.x

[17] Cavalheiro, E. A., Naffah-Mazzacoratti, M. G., Mello, L. E., Leite, J. P. (2006) The pilocarpine model of seizures. Models of seizures and epilepsy 433–448.

[18] Chen P, Chen F, Wu Y, Zhou B (2021) New insights into the role of aberrant hippocampal neurogenesis in epilepsy. Front Neurol 12:727065 34975709 10.3389/fneur.2021.727065 PMC8714646

[19] Chen S, Xu D, Fan L, Fang Z, Wang X, Li M (2022) Roles of N-methyl-D-aspartate receptors (NMDARs) in epilepsy. Front Mol Neurosci 14:797253 35069111 10.3389/fnmol.2021.797253 PMC8780133

[20] Cho GM, Lee SY, Park JH, Kim MJ, Park KJ, Choi BT, Shin HK (2020) Photobiomodulation using a low-level light-emitting diode improves cognitive dysfunction in the 5XFAD mouse model of Alzheimer’s disease. The Journal of Gerontology: Series A 75(4):631–639 10.1093/gerona/gly240 30346494

[21] Cotzias GC, Tang LC, Miller ST, Sladic-Simic D, Hurley LS (1972) A mutation influencing the transportation of manganese, L-dopa, and L-tryptophan. Science 176(4033):410–412 5026160 10.1126/science.176.4033.410

[22] Council for International Organizations of Medical Sciences (1985) International guiding principles for biomedical research involving animals. Altern Lab Anim 12:ii 11653736

[23] Crowther LM, Mathis D, Poms M, Plecko B (2019) New insights into human lysine degradation pathways with relevance to pyridoxine-dependent epilepsy due to antiquitin deficiency. J Inherit Metab Dis 42(4):620–628 30767241 10.1002/jimd.12076

[24] Curia G, Longo D, Biagini G, Jones RS, Avoli M (2008) The pilocarpine model of temporal lobe epilepsy. J Neurosci Methods 172(2):143–157 18550176 10.1016/j.jneumeth.2008.04.019 PMC2518220

[25] Curia G, Lucchi C, Vinet J, Gualtieri F, Marinelli C, Torsello A, Biagini G (2014) Pathophysiogenesis of mesial temporal lobe epilepsy: is prevention of damage antiepileptogenic? Curr Med Chem 21(6):663–688 24251566 10.2174/0929867320666131119152201 PMC4101766

[26] Dalangin R, Kim A, Campbell RE (2020) The role of amino acids in neurotransmission and fluorescent tools for their detection. Int J Mol Sci 21(17):6197 32867295 10.3390/ijms21176197 PMC7503967

[27] Disner SG, Beevers CG, Gonzalez-Lima F (2016) Transcranial laser stimulation as neuroenhancement for attention bias modification in adults with elevated depression symptoms. Brain Stimul 9(5):780–787 27267860 10.1016/j.brs.2016.05.009 PMC5007141

[28] Dubé C, Boyet S, Marescaux C, Nehlig A (2001) Relationship between neuronal loss and interictal glucose metabolism during the chronic phase of the lithium-pilocarpine model of epilepsy in the immature and adult rat. Exp Neurol 167(2):227–241 11161611 10.1006/exnr.2000.7561

[29] Engel J Jr, Kuhl DE, Phelps ME, Mazziotta JC (1982) Interictal cerebral glucose metabolism in partial epilepsy and its relation to EEG changes. Ann Neurol Off J Am Neurol Assoc Child Neurol Soc 12(6):510–517 10.1002/ana.410120603 6818896

[30] Faitg, J., Lacefield, C., Davey, T., White, K., Laws, R., Kosmidis, S., Picard, M. (2021) 3D neuronal mitochondrial morphology in axons, dendrites, and somata of the aging mouse hippocampus. Cell rep 36(6). 10.1016/j.celrep.2021.109509 PMC8423436 34380033

[31] Feng Y, Zhang C, Wei Z, Li G, Gan Y, Liu C, Deng Y (2022) Gene variations of glutamate metabolism pathway and epilepsy. Acta Epileptologica 4(1):31

[32] Fisher RS, Cross JH, French JA, Higurashi N, Hirsch E, Jansen FE, Zuberi SM (2017) Operational classification of seizure types by the International League Against Epilepsy: Position Paper of the ILAE Commission for Classification and Terminology. Epilepsia 58(4):522–530 28276060 10.1111/epi.13670

[33] Frankowski, D. W., Ferrucci, L., Arany, P. R., Bowers, D., Eells, J. T., Gonzalez-Lima, F., Lakatta, E. G. (2025) Light buckets and laser beams: mechanisms and applications of photobiomodulation (PBM) therapy. GeroSci 1–13. 10.1007/s11357-025-01505-z PMC12181550 39826026

[34] Gao J, Wang D, Zhu C, Wang J, Wang T, Xu Y, Wang Y (2024) 1H-MRS reveals abnormal energy metabolism and excitatory-inhibitory imbalance in a chronic migraine-like state induced by nitroglycerin in mice. J Headache Pain 25(1):163 39350002 10.1186/s10194-024-01872-6 PMC11441011

[35] Hanada T (2020) Ionotropic glutamate receptors in epilepsy: a review focusing on AMPA and NMDA receptors. Biomolecules 10(3):464 32197322 10.3390/biom10030464 PMC7175173

[36] Holmes E, Barrett DW, Saucedo CL, O’Connor P, Liu H, Gonzalez-Lima F (2019) Cognitive enhancement by transcranial photobiomodulation is associated with cerebrovascular oxygenation of the prefrontal cortex. Front Neurosci 13:1129 31680847 10.3389/fnins.2019.01129 PMC6813459

[37] Hong N, Kim HJ, Kang K, Park JO, Mun S, Kim HG, Ahn JC (2023) Photobiomodulation improves the synapses and cognitive function and ameliorates epileptic seizure by inhibiting downregulation of Nlgn3. Cell Biosci 13(1):8 36635704 10.1186/s13578-022-00949-6 PMC9837965

[38] Hörster F, Schwab MA, Sauer SW, Pietz J, Hoffmann GF, Okun JG, Kins S (2006) Phenylalanine reduces synaptic density in mixed cortical cultures from mice. Pediatr Res 59(4):544–548 16549526 10.1203/01.pdr.0000203091.45988.8d

[39] Hu RQ, Davies JA (1997) Glutamate receptor antagonists reduce spontaneous epileptiform activity in cortical wedges prepared from DBA/2 mice. Exp Brain Res 115:311–318 9224858 10.1007/pl00005699

[40] Kann O, Kovács R, Njunting M, Behrens CJ, Otáhal J, Lehmann TN, Heinemann U (2005) Metabolic dysfunction during neuronal activation in the ex vivo hippocampus from chronic epileptic rats and humans. Brain 128(10):2396–2407 15958506 10.1093/brain/awh568

[41] Khuman J, Zhang J, Park J, Carroll JD, Donahue C, Whalen MJ (2012) Low-level laser light therapy improves cognitive deficits and inhibits microglial activation after controlled cortical impact in mice. J Neurotrauma 29(2):408–417 21851183 10.1089/neu.2010.1745 PMC3261787

[42] Kuhl DE, Engel J Jr, Phelps ME, Selin C (1980) Epileptic patterns of local cerebral metabolism and perfusion in humans determined by emission computed tomography of 18FDG and 13NH3. Annals of Neurology: Official Journal of the American Neurological Association and the Child Neurology Society 8(4):348–360 10.1002/ana.410080403 6776878

[43] Li D, Li S, Pan M, Li Q, Song J, Zhang R (2024) The role of extracellular glutamate homeostasis dysregulated by astrocyte in epileptic discharges: a model evidence. Cogn Neurodyn 18(2):485–502 38699615 10.1007/s11571-023-10001-z PMC11061099

[44] Li X, Yang C, Shi Y, Guan L, Li H, Li S, Lin J (2021) Abnormal neuronal damage and inflammation in the hippocampus of kainic acid-induced epilepsy mice. Cell Biochem Funct 39(6):791–801 34057222 10.1002/cbf.3651

[45] Li ZY, Zheng XY, Gao XX, Zhou YZ, Sun HF, Zhang LZ, Qin XM (2010) Study of plasma metabolic profiling and biomarkers of chronic unpredictable mild stress rats based on gas chromatography/mass spectrometry. Rapid Commun Mass Spectrom 24(24):3539–3546 21080506 10.1002/rcm.4809

[46] Liu H, Zhang S, Zhang L (2021) Epileptiform activity in mouse hippocampal slices induced by moderate changes in extracellular Mg 2+, Ca 2+, and K+. BMC Neurosci 22:1–18 34301200 10.1186/s12868-021-00650-3 PMC8305515

[47] Lopes MW, Soares FMS, de Mello N, Nunes JC, Cajado AG, de Brito D, Leal RB (2013) Time-dependent modulation of AMPA receptor phosphorylation and mRNA expression of NMDA receptors and glial glutamate transporters in the rat hippocampus and cerebral cortex in a pilocarpine model of epilepsy. Exp Brain Res 226:153–163 23392471 10.1007/s00221-013-3421-8

[48] O’Brien JA, Austin PJ (2019) Effect of photobiomodulation in rescuing lipopolysaccharide-induced dopaminergic cell loss in the male Sprague-Dawley rat. Biomolecules 9(8):381 31430990 10.3390/biom9080381 PMC6723099

[49] O’Donnell CM, Barrett DW, Fink LH, Garcia-Pittman EC, Gonzalez-Lima F (2022) Transcranial infrared laser stimulation improves cognition in older bipolar patients: proof of concept study. J Geriatr Psychiatry Neurol 35(3):321–332 33525934 10.1177/0891988720988906

[50] Ooi QY, Qin X, Yuan Y, Zhang X, Yao Y, Hao H, Li L (2023) Alteration of excitation/inhibition imbalance in the hippocampus and amygdala of drug-resistant epilepsy patients treated with acute vagus nerve stimulation. Brain Sci 13(7):976 37508908 10.3390/brainsci13070976 PMC10377456

[51] Pena, I. A., Marques, L. A., Laranjeira, Â. B., Yunes, J. A., Eberlin, M. N., MacKenzie, A., Arruda, P. (2017) Mouse lysine catabolism to aminoadipate occurs primarily through the saccharopine pathway; implications for pyridoxine dependent epilepsy (PDE). Biochim Biophys Acta (BBA)-Mol Basis Dis 1863(1):121–128. 10.1016/j.bbadis.2016.09.006 27615426

[52] Pires, G., Leitner, D., Drummond, E., Kanshin, E., Nayak, S., Askenazi, M., Devinsky, O. (2021). Proteomic differences in the hippocampus and cortex of epilepsy brain tissue. Brain Commun 3(2):fcab021. 10.1093/braincomms/fcab021 PMC8214864 34159317

[53] Rheims S, Represa A, Ben-Ari Y, Zilberter Y (2008) Layer-specific generation and propagation of seizures in slices of developing neocortex: role of excitatory GABAergic synapses. J Neurophysiol 100(2):620–628 18497363 10.1152/jn.90403.2008

[54] Rojas JC, Gonzalez-Lima F (2013) Neurological and psychological applications of transcranial lasers and LEDs. Biochem Pharmacol 86(4):447–457 23806754 10.1016/j.bcp.2013.06.012

[55] Rumping L, Vringer E, Houwen RH, van Hasselt PM, Jans JJ, Verhoeven-Duif NM (2020) Inborn errors of enzymes in glutamate metabolism. J Inherit Metab Dis 43(2):200–215 31603991 10.1002/jimd.12180 PMC7078983

[56] Salehpour F, Farajdokht F, Erfani M, Sadigh-Eteghad S, Shotorbani SS, Hamblin MR, Mahmoudi J (2018) Transcranial near-infrared photobiomodulation attenuates memory impairment and hippocampal oxidative stress in sleep-deprived mice. Brain Res 1682:36–43 29307593 10.1016/j.brainres.2017.12.040 PMC5801165

[57] Salehpour F, Mahmoudi J, Kamari F, Sadigh-Eteghad S, Rasta SH, Hamblin MR (2018) Brain photobiomodulation therapy: a narrative review. Mol Neurobiol 55:6601–6636 29327206 10.1007/s12035-017-0852-4 PMC6041198

[58] Sancho-Vaello E, Marco-Marín C, Gougeard N, Fernández-Murga L, Rüfenacht V, Mustedanagic M, Häberle J (2016) Understanding N-acetyl-L-glutamate synthase deficiency: mutational spectrum, impact of clinical mutations on enzyme functionality, and structural considerations. Hum Mutat 37(7):679–694 27037498 10.1002/humu.22995

[59] Scheffer IE, Berkovic S, Capovilla G, Connolly MB, French J, Guilhoto L, Zuberi SM (2017) ILAE classification of the epilepsies: Position paper of the ILAE Commission for Classification and Terminology. Epilepsia 58(4):512–521 28276062 10.1111/epi.13709 PMC5386840

[60] Sierra A, Gröhn O, Pitkänen A (2015) Imaging microstructural damage and plasticity in the hippocampus during epileptogenesis. Neuroscience 309:162–172 25934032 10.1016/j.neuroscience.2015.04.054

[61] Sirven, J. I. (2015) Epilepsy: a spectrum disorder. Cold Spring Harbor perspectives in med 5(9). 10.1101/cshperspect.a022848 PMC4561391 26328931

[62] Smeland OB, Hadera MG, McDonald TS, Sonnewald U, Borges K (2013) Brain mitochondrial metabolic dysfunction and glutamate level reduction in the pilocarpine model of temporal lobe epilepsy in mice. J Cereb Blood Flow Metab 33(7):1090–1097 23611869 10.1038/jcbfm.2013.54 PMC3705438

[63] Team RC (2013) R: a language and environment for statistical computing. R foundation for statistical computing

[64] Tsai CM, Chang SF, Li CC, Chang H (2022) Transcranial photobiomodulation (808 nm) attenuates pentylenetetrazole-induced seizures by suppressing hippocampal neuroinflammation, astrogliosis, and microgliosis in peripubertal rats. Neurophotonics 9(1):015006–015006 35345494 10.1117/1.NPh.9.1.015006 PMC8955735

[65] Turski WA, Cavalheiro EA, Schwarz M, Czuczwar SJ, Kleinrok Z, Turski L (1983) Limbic seizures produced by pilocarpine in rats: behavioural, electroencephalographic and neuropathological study. Behav Brain Res 9(3):315–335 6639740 10.1016/0166-4328(83)90136-5

[66] Vargas E, Barrett DW, Saucedo CL, Huang LD, Abraham JA, Tanaka H, Gonzalez-Lima F (2017) Beneficial neurocognitive effects of transcranial laser in older adults. Lasers Med Sci 32(5):1153–1162 28466195 10.1007/s10103-017-2221-y PMC6802936

[67] van Liempd SM, Cabrera D, Lee FY, González E, Dell’Angelica EC, Ghiani CA, Falcon-Perez JM (2017) BLOC-1 deficiency causes alterations in amino acid profile and in phospholipid and adenosine metabolism in the postnatal mouse hippocampus. Sci Rep 7(1):5231 28701731 10.1038/s41598-017-05465-z PMC5507893

[68] Vogel DD, Ortiz-Villatoro NN, de Freitas L, Aimbire F, Scorza FA, Albertini R, Scorza CA (2021) Repetitive transcranial photobiomodulation but not long-term omega-3 intake reduces epileptiform discharges in rats with stroke-induced epilepsy. J Biophotonics 14(1):e202000287 32888387 10.1002/jbio.202000287

[69] Wade ZS, Barrett DW, Davis RE, Nguyen A, Venkat S, Gonzalez-Lima F (2023) Histochemical mapping of the duration of action of photobiomodulation on cytochrome c oxidase in the rat brain. Front Neurosci 17:1243527 37700747 10.3389/fnins.2023.1243527 PMC10493319

[70] Wu Y, Ma Y, Li Q, Li J, Zhang D, Zhang Y, Xue M (2024) Multi-omics analysis reveals phenylalanine enhance mitochondrial function and hypoxic endurance via LKB1/AMPK activation. J Transl Med 22(1):920 39390477 10.1186/s12967-024-05696-5 PMC11465566

[71] Zhao S, Khoo S, Ng SC, Chi A (2022) Brain functional network and amino acid metabolism association in females with subclinical depression. Int J Environ Res Public Health 19(6):3321 35329007 10.3390/ijerph19063321 PMC8951207

